# Use of sequence analysis for classifying individual antidepressant trajectories to monitor population mental health

**DOI:** 10.1186/s12888-020-02952-y

**Published:** 2020-11-23

**Authors:** Mark Cherrie, Sarah Curtis, Gergő Baranyi, Stuart McTaggart, Niall Cunningham, Kirsty Licence, Chris Dibben, Clare Bambra, Jamie Pearce

**Affiliations:** 1 School of GeoSciences, The University of Edinburgh, Edinburgh, Scotland, UK; 2 Institute of Occupational Medicine, Edinburgh, Scotland, UK; 3 Department of Geography, Durham University, Durham, UK; 4 Public Health Scotland, Edinburgh, UK; 5 School of Geography, Politics & Sociology, Newcastle University, Newcastle upon Tyne, UK; 6 Health Protection Scotland, Glasgow, UK; 7 Scottish Centre for Administrative Data Research, University of Edinburgh, Edinburgh, UK; 8 Population Health Sciences Institute, Newcastle University, Newcastle upon Tyne, UK

**Keywords:** Public health monitoring, Health service use, Administrative data, Prescriptions, Antidepressants, Mental health, Sequence analysis

## Abstract

**Background:**

Over the past decade, antidepressant prescriptions have increased in European countries and the United States, partly due to an increase in the number of new cases of mental illness. This paper demonstrates an innovative approach to the classification of population level change in mental health status, using administrative data for a large sample of the Scottish population. We aimed to identify groups of individuals with similar patterns of change in pattern of prescribing, validate these groups by comparison with other indicators of mental illness, and characterise the population most at risk of increasing mental ill health.

**Methods:**

National Health Service (NHS) prescription data were linked to the Scottish Longitudinal Study (SLS), a 5.3% sample of the Scottish population (*N* = 151,418). Antidepressant prescription status over the previous 6 months was recorded for every month for which data were available (January 2009–December 2014), and sequence dissimilarity was computed by optimal matching. Hierarchical clustering was used to create groups of participants who had similar patterns of change, with multi-level logistic regression used to understand group membership.

**Results:**

Five distinct prescription pattern groups were observed, indicating: no prescriptions (76%), occasional prescriptions (10%), continuation of prior use of prescriptions (8%), a new course of prescriptions started (4%) or ceased taking prescriptions (3%). Young, white, female participants, of low social grade, residing in socially deprived neighbourhoods, living alone, being separated/divorced or out of the labour force, were more likely to be in the group that started a new course of antidepressant prescriptions.

**Conclusions:**

The use of sequence analysis for classifying individual antidepressant trajectories offers a novel approach for capturing population-level changes in mental health risk. By classifying individuals into groups based on their anti-depressant medication use we can better identify how over time, mental health is associated with individual risk factors and contextual factors at the local level and the macro political and economic scale.

## Background

In recent years there has been a global increase in mental health morbidity [1], an increase in treatment by antidepressant medicines in high income counties [2] and a growing awareness of gaps in service delivery and resources in low and middle income countries [3]. For example, between 2000 and 2015 antidepressant use more than doubled in some countries including the UK, Germany, Sweden, Australia [4]. A similar trend has been observed in the United States, where between 1999 and 2010 there was a 63% increase in reported antidepressant medication use [5]. In Scotland, over a 12 month period during 2012/2013, the number of antidepressant items recorded was 5.2 million, dispensed to 747,158 patients in Scotland, costing £29.5 million; a 52% increase from 10 years previously [6] . At this time, 9% of the Scottish population reported at least two depressive symptoms [7] and 12% took an antidepressant every day.

Population-level administrative data linked with health services information on prescriptions for mental illness offer potential to understand predictors of mental illness over time Ecological studies have captured change in mental health using fixed effects models of suicide rates, self-report and antidepressant use [8–10]. Repeated cross-sectional analysis of self-reported mental illness (e.g. General Health Questionnaire) are also common, although measures were analysed dichotomously [11, 12]. These methods make it difficult to distinguish trajectories of mental illness among groups in the population. Using administrative data linked with individual prescription use may be a way to address these limitations. A descriptive analysis of national changes in *aggregated* antidepressant use in Scotland has been conducted for 1995–2007 [10]. Studies using individual-level data on antidepressant use usually concentrate on ‘any antidepressant use’ [13] or ‘chronic’ use [14], but not on occasional, increasing or decreasing use, which are harder to define, but may be more indicative of change in population mental health.

We present a novel method for distinguishing changes in prescription use at the individual level in a very large population sample. In particular, we demonstrate the use of sequence analysis of longitudinal data to define groups of people who have shared a similar pattern of change. By analysing how antidepressant trajectories relate to other indicators of mental illness, we examine the validity of trajectories as a measure of mental illness. By modelling prescription trajectories in relation to individual and neighbourhood risk factors we also show how we can better understand the inequalities in the experiences of mental illness.

## Methods

### Study sample

We used data drawn from the Scottish Longitudinal Study (SLS), a population representative sample (5.3%) of individuals living in Scotland, drawn from the population census. The SLS sample are selected as having one of 20 semi-random birth dates and enter the study at one of the 1991, 2001 and 2011 censuses or through being a new birth or immigrant. To add data on NHS service use and prescriptions for mental health conditions for 2009–2014 to the SLS sample, SLS staff used the SLS/CHI (Community Health Index – population index used for healthcare purposes) number. (This is based on the Community Health Index; an administrative index used for healthcare purposes). NHS Service use data for linkage were extracted in secure conditions by staff at the electronic Data Research & Innovation Service (eDRIS), at the Information Services Division (ISD) of NHS National Services Scotland. The SLS and NHS data extracts were provided anonymized for our analyses, which were undertaken in a secure and carefully regulated setting. Results were assessed by SLS staff prior to publication to protect the anonymity of the individuals in the data set.

### Antidepressant trend groups

Prescription data were originally derived from the Prescription Information System by NHS Scotland [15], which collects information on medication prescribed and dispensed at the community level. The criteria for mental illness related prescriptions was defined by the British National Formulary (BNF) numbers. This provided three classes of drugs: antidepressants (BNF: 4.3), anxiolytics (BNF: 4.1.2) and antipsychotics (BNF: 4.2), of which antidepressants were the main focus for the current study. Antidepressants have been shown to be prescribed conservatively in Scotland, when there is good reason to suggest the patient is experiencing depression [16]. Using information extracted from prior research using text-mining of dose instructions [17], we discounted Amitriptyline and Notriptyline at low doses (≤ 30 mg per day; i.e. three doses of the 10 mg tablet or one 25 mg tablet) from our antidepressant dataset, since these medicines are often used at low dose for non-mental illness related conditions (e.g. neuropathic pain) [18]. In order to measure prescription status over time, for each month, we calculated whether the participant had been prescribed antidepressants in the preceding 6 months from 2009 to 2014. Six months was chosen because it corresponds to recommended course of initial treatment to treat clinical depression under NHS guidelines [19]. Using the TraMineR package in R [20], we computed sequence dissimilarity using optimal matching (i.e. minimising insertion, deletion, substitution costs) and applied hierarchical clustering to produce five groupings of individuals by ‘antidepressant prescription trend’.

### Relationship between antidepressant prescription trend group and auxiliary indicators of mental illness

In order to understand how auxiliary indicators of mental illness associated with the antidepressant trends we used measures of mental illness before and during the time prescriptions data was recorded. A further linkage was made to information drawn from NHS General Inpatient Hospital services administrative data, which indicated use of other services for mental health conditions (ICD10 classified; F10-F48) (SMR 04; Mental Health Inpatient & Day Case). Data on inpatient use of hospital services was used from 2001 to 2008, as a marker of mental illness before the start of the SLS member’s prescription record. A final indicator of participant mental illness was derived from a question SLS members were asked in the 2011 census: ‘Do you have any of the following conditions which have lasted, or are expected to last, at least 12 months?’, in which they could answer as part of a multiple choice response: ‘Mental Health condition’. This provides a self-reported mental health indicator at one time point, mid-way through the period covered by the prescription data. Data on anxiolytics and antipsychotics prescriptions were also used, with participants defined as ‘being prescribed’ if they had been prescribed anxiolytics and antipsychotics on at least one occasion from 2009 to 2014.

A series of multilevel logistic regression models were developed to determine the association between each antidepressant trajectory (i.e. dependent variables) and each of the auxiliary measures of mental illness - hospital admissions, self-reported mental illness, any anxiolytic prescriptions, and any antipsychotic prescriptions (i.e. independent variables). Additional covariates in these models included age, sex, Carstairs deprivation, employment status, ethnicity, social grade, living alone, and marital status, which are explained in full in the next section. Local authority of residence in 2011 was used as a random effect. These analyses were conducted in R version 3.4.0, using the ‘lme4’ package. We report odd ratios (OR) and 95% confidence intervals (CI).

### Relationship between antidepressant prescription trend group and individual and neighbourhood risk factors

We used a number of variables describing individual attributes of sample members that were derived from the SLS census data. These included demographic characteristics such as sex, age (in 2011) and ethnicity, indicated in the literature as relevant to risks for mental illness needing prescription. Females have a 40% higher risk of antidepressant use [21]. Mental health conditions treated by antidepressants increase especially as people reach their 20’s, with people in their middle years (50–54 years old) most likely to be patients treated with antidepressants [22]. Antidepressant use tends to be lower in black and minority ethnic groups [23]. Ethnicity was classified as White/non-White/missing to reduce the risk of disclosure from low numbers in some categories (e.g. Black). We used two variables on living arrangements likely to be relevant to risk for depression: marital status in 2011 (“single”, “married”, “separated”, “divorced”, “widowed”) and whether the participant was living alone in 2011 (“Not living alone”, “Living Alone”). Antidepressant use is more likely to occur in recently divorced individuals [24] and those of working age and living alone were found to have an 81% higher risk of antidepressant use over a 7 year period [25]. Socioeconomic indicators included the employment status.. This was categorized as: “In employment”; “Unemployed”; “Retired”; “Out of labour force” (all others who were economically inactive, including those who were students, looking after home or family, long-term sick or disabled or other). We also used an approximation of an individual’s social grade was based on the socio-economic classification used by the Market Research and Marketing Industries (I-high to V-low). Local area deprivation was measured using a census-based composite indicator of various aspects of socio-economic deprivation (Carstairs decile). Carstairs Decile was measured at the level of Census Output Areas in 2011 (*n* = 46,351; mean population = 114), which approximate to the person’s neighbourhood of residence.. Carstairs index is comprised of the sum of four z-scored components: male unemployment rate, lack of car ownership, overcrowding and low social class (IV and V). Neighbourhood income deprivation is known to be significantly and independently associated with antidepressant use in Scotland [26].

As in the first set of models (i.e. auxiliary indicators of mental illness), membership of each antidepressant prescription trajectory group was used as the dependent variable. In these models we have omitted the auxiliary indicators of mental illness.

## Results

### Antidepressant prescription trend groups

**Fig. 1:**
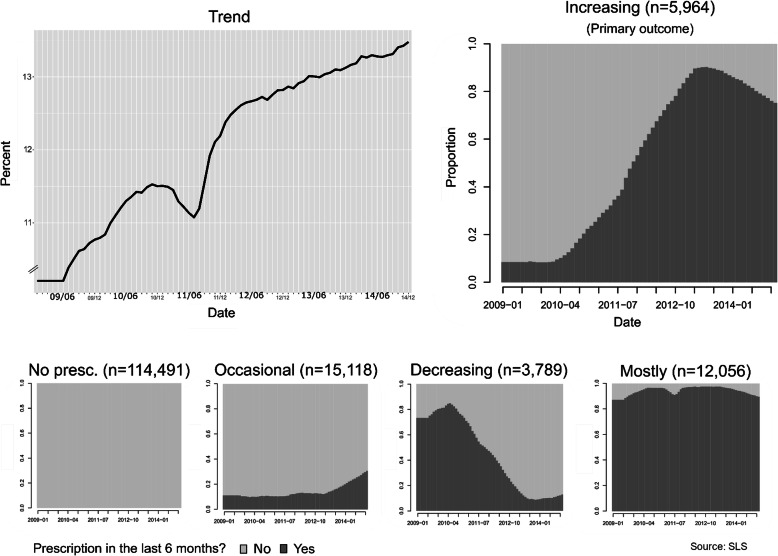
Antidepressant prescription trend and groups

The percentage of the individuals who had been prescribed antidepressants at some point from June 2009 (i.e. 09/06) to December 2014 (i.e. 14/12) is presented in Fig. 1. This shows that antidepressant prescription use has increased in Scotland over the time period; between June 2009 and December 2014 the percentage of the population which had a prescription in the previous 6 months increased from 10.5 to 13.4%, with a trough from March–November 2011. The trough was an artefact from data loss as a consequence of medicine shortages of one of the most widely prescribed medicines (citalopram). We classified the individual sequences into five antidepressant prescription groups; these groups are visualised as the proportion of participants prescribed antidepressants for each month in the time period (Fig. 1). These groups are briefly described below.

Two groups are considered as *not treated for persistent depression:* those who were not being prescribed any antidepressants (*N* = 114,491; 76%), and those with occasional low levels of prescriptions for antidepressants (with a slight increase since March 2013) (*N* = 15,118; 10%). A third group may comprise of individuals who were *in recovery* from depression, with decreasing levels of prescriptions since April 2010 (*N* = 3789; 3%). A fourth group could be made up of those treated for *worsening levels of depression,* having increasing trends in prescriptions since April 2010 (*N* = 5964; 4%). The fifth group are defined as probably being treated for *long-term chronic depression,* receiving prescribed continuously over the time studied (*N* = 12,056; 8%). There has been a net increase in the number of people being prescribed antidepressants (*N* = 2175; + 1.4%).

### Relationship between antidepressant prescription trend group and auxiliary indicators of mental illness

Table 1 shows the results of the 20 multivariate models with auxiliary indicators of mental illness. We found a gradient in the strength of relationship between self-reported mental illness in 2011 and antidepressant prescription group (Table 1). The relationship was similar with past hospital admission for mental health care with an exception; previous hospital admission was not associated with increasing prescription use (OR 1.13; 95%CI 0.86–1.41). Prescription for anxiolytics was linearly associated with occasional (OR 1.91; 95%CI 1.85–1.97), decreasing (OR 2.58; 95%CI 2.48–2.67), increasingly (OR 3.28; 95%CI 3.20–3.35) and mostly on antidepressant prescriptions (OR 4.91; 95%CI 4.85–4.96). In comparison, antipsychotics were more likely for those whose antidepressant prescription pattern was defined as ‘occasional’ (OR 1.21; 95%CI 1.11–1.31), equally more likely for those with ‘increasing’ (OR 2.12; 95%CI 2.48–2.67) or ‘decreasing’ (OR 2.17; 95%CI 2.05–2.29) antidepressant use, and much more likely for those mostly on antidepressants (OR 6.16; 95%CI 6.08–6.24).

**Table 1:** Association between indicators of mental illness and antidepressant prescription group

		Antidepressant Prescription Group				
Variable	Reference	No prescriptions OR (95% CI)	Occasional OR (95% CI)	Decreasing OR (95% CI)	Increasing OR (95% CI)	Mostly OR (95% CI)
Self-reported mental illness in 2011	No self-reported mental illness in 2011	0.09 (0.02, 0.15)	0.84 (0.76, 0.92)	3.83 (3.74, 3.93)	1.84 (1.75, 1.93)	12.97 (12.92, 13.03)
Hospital Admission for mental health care from 2001 to 2008	No Hospital Psychiatric Admission 2001–2008	0.20 (0.03, 0.36)	0.72 (0.48, 0.96)	1.79 (1.52, 1.41)	1.13 (0.86, 1.41)	6.04 (5.89, 6.20)
Anxiolytic Prescription	No anxiolytics prescription (2009–2014)	0.13 (0.08, 0.19)	1.91 (1.85, 1.97)	2.58 (2.48, 2.67)	3.28 (3.20, 3.35)	4.91 (4.85, 4.96)
Antipsychotics Prescription	No antipsychotic prescription (2009–2014)	0.15 (0.07, 0.23)	1.21 (1.11, 1.31)	2.12 (1.98, 2.26)	2.17 (2.05, 2.29)	6.16 (6.08, 6.24)

Adjusted for age, sex, Carstairs deprivation, employment status, ethnicity, social grade, living alone, and marital status; source: SLS

### Relationship between antidepressant prescription trend group and individual and neighbourhood risk factors

Table 2 shows the results of the five multivariate multi-level logistic regression models with individual and neighbourhood risk factors. Being female was associated with higher likelihood of being in the prescription groups, especially being prescribed most of the time (OR 2.19; 95% CI 2.15–2.24) (Table 2). Age in 2011 had a varied relationship with the prescription groups, generally being between 26 and 45 years old was associated with increasing prescriptions. ‘White’ ethnicity was associated with a higher likelihood of being prescribed; ‘non-white’ ethnicity was inversely associated with increasing prescriptions in particular (OR 0.47; 0.16–0.79). Being separated or divorced was associated with having been prescribed antidepressants; being separated had a stronger relationship with decreasing prescriptions (OR 1.72; 95% CI 1.58–1.87), whilst receiving occasional prescriptions was more strongly associated with being divorced (OR 1.31; 95% CI 1.24–1.37). Living alone was associated with higher likelihood of being prescribed, especially being prescribed most of the time (OR 1.28; 1.22–1.34), but not for those with occasional prescriptions (OR 0.99; 0.94–1.11). Being out of the labour force was the strongest predictor tested, with a 4-fold higher likelihood of being prescribed most of the time (OR 4.05; 4.01–4.11), being unemployed was associated with higher likelihood of decreasing prescriptions (OR 2.09; 1.93–2.24). Generally, lower social grade and higher Carstairs neighbourhood deprivation were associated with a higher likelihood of being prescribed, with the strongest relationships with being prescribed most of the time.

**Table 2:** Demographic, living arrangement and socioeconomic predictors of antidepressant prescription group membership

		Antidepressant Prescription Group				
Variable	Reference	No prescriptions	Occasional	Decreasing	Increasing	Mostly
		OR (95% CI)	OR (95% CI)	OR (95% CI)	OR (95% CI)	OR (95% CI)
Sex (Female)	Male	0.49	1.53	1.76	1.80	2.19
		(0.46, 0.52)	(1.49, 1.57)	(1.69, 1.83)	(1.75, 1.86)	(2.15, 2.24)
Age (36:45)	26:35 (in 2011)	0.92	0.82	1.08	0.99	1.86
		(0.88, 0.97)	(0.76, 0.88)	(0.96, 1.19)	(0.90, 1.07)	(1.78, 1.95)
Age (46:55)		0.91	0.79	0.92	0.85	2.32
		(0.86, 0.95)	(0.73, 0.85)	(0.80, 1.04)	(0.76, 0.94)	(2.24, 2.40)
Age (56:65)		1.2	0.61	0.87	0.61	1.97
		(1.15, 1.25)	(0.54, 0.68)	(0.73, 1.00)	(0.50, 0.72)	(1.88, 2.06)
Age (66:75)		1.53	0.58	0.72	0.55	1.36
		(1.47, 1.60)	(0.49, 0.67)	(0.54, 0.90)	(0.40, 0.70)	(1.25, 1.47)
Age (76:85)		1.47	0.75	0.74	0.62	1.04
		(1.40, 1.55)	(0.65, 0.86)	(0.54, 0.94)	(0.45, 0.79)	(0.91, 1.16)
Age (86+)		1.57	0.80	0.94	0.66	0.67
		(1.47, 1.68)	(0.66, 0.95)	(0.68, 1.19)	(0.43, 0.89)	(0.48, 0.86)
Ethnicity (Non-White)	White	1.72	0.86	0.75	0.47	0.49
		(1.60, 1.85)	(0.70, 1.02)	(0.42, 1.08)	(0.16, 0.79)	(0.26, 0.71)
Ethnicity (Missing)		0.93	1.12	1.07	1.07	0.97
		(0.86, 1.00)	(1.02, 1.22)	(0.89, 1.26)	(0.92, 1.22)	(0.85, 1.08)
Marital Status (married)	Single	1.01	1.01	0.90	1.02	0.96
		(0.97, 1.05)	(0.95, 1.06)	(0.80, 1.01)	(0.94, 1.10)	(0.90, 1.03)
Marital Status (separated)		0.56	1.5	1.72	1.43	1.39
		(0.50, 0.63)	(1.42, 1.59)	(1.58, 1.87)	(1.30, 1.55)	(1.30, 1.49)
Marital Status (divorced)		0.71	1.31	1.28	1.22	1.23
		(0.67, 0.76)	(1.24, 1.37)	(1.16, 1.40)	(1.12, 1.32)	(1.15, 1.30)
Marital Status (widowed)		0.98	1.03	1.07	0.98	0.99
		(0.92, 1.04)	(0.95, 1.11)	(0.92, 1.21)	(0.85, 1.10)	(0.90, 1.07)
Living Alone	Not living alone	0.87	0.99	1.18	1.10	1.28
		(0.83, 0.91)	(0.94, 1.04)	(1.09, 1.28)	(1.02, 1.18)	(1.22, 1.34)
Social Grade (II)	Grade I	0.78	1.24	1.14	1.22	1.26
	(Professional)	(0.74, 0.82)	(1.19, 1.30)	(1.03, 1.25)	(1.13, 1.30)	(1.19, 1.33)
Social Grade (III)		0.75	1.33	1.16	1.17	1.32
		(0.71, 0.79)	(1.27, 1.39)	(1.04, 1.28)	(1.08, 1.26)	(1.25, 1.40)
Social Grade (IV)		0.66	1.41	1.26	1.37	1.48
		(0.61, 0.70)	(1.35, 1.47)	(1.15, 1.38)	(1.27, 1.46)	(1.41, 1.55)
Social Grade (V)		0.59	1.31	1.35	1.36	1.69
		(0.52, 0.66)	(1.22, 1.41)	(1.19, 1.51)	(1.23, 1.50)	(1.59, 1.78)
Employment (Retired)	Employed	0.65	1.1	1.51	1.23	1.96
		(0.60, 0.70)	(1.02, 1.17)	(1.38, 1.64)	(1.12, 1.35)	(1.89, 2.03)
Employment (Out of labour force)		0.34 (0.31, 0.38)	1.25 (1.19, 1.30)	2.23 (2.14, 2.32)	1.75 (1.67, 1.82)	4.06 (4.01, 4.11)
Employment (Unemployed)		0.53	1.73	2.09	1.54	1.42
		(0.46, 0.60)	(1.64, 1.82)	(1.93, 2.24)	(1.40, 1.67)	(1.30, 1.54)
Carstairs (decile 2)	Decile 1	0.93	1.06	1.08	1.02	1.09
	(Affluent)	(0.87, 0.99)	(0.98, 1.14)	(0.92, 1.24)	(0.90, 1.15)	(0.99, 1.18)
Carstairs (decile 3)		0.90	1.06	1.00	1.10	1.19
		(0.84, 0.96)	(0.98, 1.14)	(0.84, 1.16)	(0.97, 1.22)	(1.09, 1.28)
Carstairs (decile 4)		0.80	1.16	1.32	1.13	1.27
		(0.74, 0.86)	(1.08, 1.24)	(1.17, 1.48)	(1.01, 1.25)	(1.18, 1.37)
Carstairs (decile 5)		0.80	1.15	1.26	1.22	1.27
		(0.74, 0.86)	(1.07, 1.23)	(1.11, 1.42)	(1.09, 1.34)	(1.17, 1.36)
Carstairs (decile 6)		0.74	1.27	1.31	1.1	1.44
		(0.68, 0.79)	(1.19, 1.35)	(1.16, 1.47)	(0.97, 1.23)	(1.34, 1.53)
Carstairs (decile 7)		0.70	1.25	1.34	1.34	1.50
		(0.64, 0.75)	(1.17, 1.33)	(1.18, 1.49)	(1.22, 1.47)	(1.41, 1.60)
Carstairs (decile 8)		0.64	1.37	1.44	1.30	1.59
		(0.59, 0.70)	(1.29, 1.45)	(1.29, 1.59)	(1.18, 1.42)	(1.50, 1.68)
Carstairs (decile 9)		0.61	1.40	1.37	1.35	1.72
		(0.55, 0.67)	(1.32, 1.48)	(1.21, 1.52)	(1.22, 1.47)	(1.62, 1.81)
Carstairs (decile 10)		0.54	1.54	1.54	1.52	1.74
		(0.48, 0.60)	(1.46, 1.63)	(1.38, 1.70)	(1.40, 1.65)	(1.64, 1.83)

Source: SLS

## Discussion

### Main findings

We have demonstrated an approach to monitoring change in population mental health indicated by antidepressant prescriptions. We found the strength of the association between demographic, living arrangement, socioeconomic factors and antidepressant use varies by the pattern of prescription use over a six-year period. Compared with those receiving ‘no prescriptions’ other groups showed similar relative risks in association with variables such as sex, social grade and neighbourhood deprivation. However, the relative risk of ‘increasing’ prescription use showed more distinct associations with variables describing age, living alone, ethnicity, marital status and employment status. Young, white, female participants, of low social grade, living in deprived neighbourhoods, living alone, being separated/divorced or out of the labour force, were more likely to have started using antidepressants during the study period. This may be because of the social and economic climate at this time, which included a range of austerity-related measures that had a disproportionate impact on these women. Research has estimated that 85% of tax and benefit changes have impacted on women’s incomes – particularly on low-income women living in deprived areas [27]. Furthermore, public sector service cuts (such as to libraries, children’s centres, community centres, advice services etc.) have adversely affected women – because women are higher users of these local resources and are more likely to be employed in the public and voluntary sector than men are (ibid.). They are also more likely to be engaged in low-wage work, and have more sustained engagement with the benefit system. This has led to discussions about how women have experienced a ‘triple jeopardy’ of public sector service reductions, job losses and welfare reform [28].

### Strengths

The use of 6 years of monthly prescription data in a large representative sample of the Scottish population is a major strength of the study. The declining stigma surrounding treatment and increasing awareness of mental health may strengthen this indicator of mental illness in the future [29]. The auxiliary measures of mental illness history (admissions) and subjective mental illness status are also major strengths. This is one of the few large studies that has linked multiple indicators of mental illness, which is key to understanding a complete picture of population mental health [30]. Administrative data does not suffer from loss to follow up to the same extent as cohort studies that rely on non-routine methods of follow up, although there is some attrition due to death and migration. We have applied a novel technique to classify six-month prescription status. Previously this method has had limited application in health studies, with patterns of health care access a recent exception [31]. This technique has the advantage of uncovering the complexity of being prescribed medication and the pattern of relapse or remission that corresponds to disease progression, which is obscured by more commonly applied dichotomous measures.

### Weaknesses

The prescriptions data were not available before 2009, so we were unable to compare with usage during this time, which might have meant that some of the participants classified as having ‘increasing’ prescription use had been prescribed previously. Information on daily defined dose was not available. There were changes to the cost of treatment during the study period, whereby prescription charges were abolished in from the 1st of April 2011, which might have had an impact on poorer individuals seeking help. We did not have the medical diagnosis so a small number of the prescriptions may have been used to treat conditions other than depression. We did not consider co-morbidities, as this was out of the scope of the study. We also assumed that treatment dispensing was synonymous with antidepressant use, but we did not have information on actual consumption to verify this. Whilst we have named antidepressant groups based on trajectory at a population level there is likely to be significant heterogeneity in individual circumstances, whereby individuals are not experiencing the group level dynamic, e.g. co-morbidities and interactions with anxiolytic and anti-psychotic treatments might be driving antidepressant trend rather than the disease worsening. Covariate information was limited to measures collected in the census, therefore we were unable to understand effect of other lifetime factors [32] shown to be important for predicting antidepressant prescriptions (e.g. tobacco consumption). Although mental health service use stigma may be declining, it still may inhibit use of mental health care by some of those in need The current results are limited in that they underestimate certain populations at risk (e.g. young males) [33]. Therefore, some of the relationships observed between covariates and antidepressant trends may reflect differences in barriers to seeking medical help [34]. We acknowledge that low multicollinearity among our covariates will have slightly decreased precision in our estimates, but not biased our results.

### Comparison with existing literature

Similar methods have been used in previous studies to classify trajectories of annual antidepressant dose over time [35]. The authors of that study use latent class model, which is shown provide similar groupings to the sequence analysis used in the current study [36]. The sample used in their study was very specific – patients before and after being granted disability pension due to common mental disorders [35]. These individuals would have formed part of our ‘out of labour force’ group, which had the strongest relationship (OR 4.06; 95% CI 4.01 to 4.11) with having been prescribed most of the time and might explain why they found homogeneity in the pattern of the Daily Defined Dose (DDD) (i.e. 89% of the sample varied very little). We have shown how the current method could provide a scalable international comparable way to monitor medication use in the general population.

The relationship between socioeconomic variables and antidepressant pattern indicates that the greatest nequality exists for long-term prescription use. Previously it was found that there was little socioeconomic patterning in antidepressant review consultations in Scotland [37], which suggests that rather than differences in healthcare provision, the difference is due to disease severity. We found that unemployment was associated with decreasing use of antidepressants similar to other studies, which have found that unemployment status correlates with decreasing antidepressant use [38]. This effect is thought to be driven by health selection, whereby mental health status deteriorates before unemployment, and then improves during unemployment [39, 40], perhaps due to relief of work related stressors. Living alone had stronger associations with antidepressants in another study (OR 1.81; 95%CI 1.46–2.23) [25] than in the current study (OR ranged from 1.10 to 1.28 for ‘decreasing’, ‘increasing’ and ‘most of the time’ groups), which may be explained by the differences in sample. The association between living alone and common mental illness is found in other research to be mostly (84%) due to a great sense of loneliness [41]. Previous work estimated that psychotropic medication peaks 6–9 months before divorce and declined for 18 months thereafter [42], however we found that separation (which often precedes divorce) had a stronger relationship with a reduction in antidepressants, indicating that separation may provide a buffer to mental health distress between marriage and divorce.

The pattern of antidepressant prescriptions gives a good indication of mental illness; being on prescriptions most of the time is strongly and positively associated with self-reported mental illness and previous hospital admissions; the inverse is true for ‘no prescriptions’ or ‘occasional’ prescriptions. ‘Increasing’ prescriptions had a weaker relationship with self-reported mental illness than decreasing prescriptions, which shows that there might be a lag between starting medication and identifying oneself as having a mental illness. No association exists between previous psychiatric hospital admissions and the ‘increasing’ prescriptions group, which may indicate that these patients have had a new episode of depression following the Recession. The negative relationship between self-reporting mental illness and previous hospital admissions, and the ‘occasional’ prescription group confirmed that this group is unlikely to be suffering from persistent depression. Significant polypharmacy existed with antidepressant, anxiolytics and antipsychotics prescription; a possible sign of comorbid mental disorders. Antidepressants combined with anxiolytics were prescribed together particularly for those who had increased their antidepressant prescriptions. Antidepressants combined with antipsychotics were prescribed especially for those that have been prescribed antidepressants continuously over the study period. Polypharmacy has been advocated as a way to treat severe and treatment-resistant depression [43], however concerns have been raised especially for antidepressant-antipsychotic combinations with benefits outweighed by the increased risk of adverse effects (e.g. suicide) [44].

### Implications for public health and research

Public health organisations could utilise the methods outlined in this paper to continuously monitor population mental health. The current application has shown the national trends and groupings for 2009–2014, but it could also be useful for a number of spatiotemporal configurations. Further drilling down to refine groupings may also be useful. Future research could usefully develop this approach to examine measures of mental illness across the life course to understand continuation, relapse and remission, in combination with personal experiences by patients [45]. In particular, a high-risk change in living arrangements - going from marriage to separation and divorce and how that can lead to loneliness associated with living alone, warrants further investigation.

## Conclusions

This study provides a novel approach to understanding population-level risk group changes in mental health, using health service (prescription) data. These methods provide new opportunities for policymakers to monitor population mental health inequalities. Combining the at-risk prescription group from the current study (i.e. white, female participants, of low social grade, living in deprived neighbourhoods, living alone, being separated/divorced or out of the labour force), with the at-risk profiles of other indicators of mental illness would improve the approach to delivering policies to help those most in need.

## Data Availability

Data sharing is not applicable to this article. The SLS data can be accessed by following the steps outlined here: https://sls.lscs.ac.uk/guides-resources/step-bystep-guide-to-accessing-sls-data-1/.

## Abbreviations

NHS: National Health Service
SLS: Scottish Longitudinal Study
eDRIS: Electronic Data Research & Innovation Service
ISD: Information Services Division
BNF: British National Formulary
ICD: International Classification of Diseases
SMR: Scottish Morbidity Record
OR: Odds Ratio
95% CI: 95% Confidence Intervals
